# Unlocking the Healthy Aging Potential of Novel NMN-Based Nutritional Formulations: Cardiopulmonary, Cognitive, and Aging-Related Outcomes in a 42-Day Open-Label Study

**DOI:** 10.3390/nu18213490

**Published:** 2026-10-23

**Authors:** Tomonari Abe, Nick Yoshida, Mohamed Abdelhakim, Hiroshi Abe

**Affiliations:** 1 Abe Yoan Aging Research Institute, Tokyo 104-0061, Japanmhmedhakim7@gmail.com (M.A.);; 2 Department of Plastic, Reconstructive and Regenerative Medicine, Nippon Medical School Hospital, Tokyo 113-8603, Japan

**Keywords:** nicotinamide mononucleotide, NMN, beta-hydroxybutyrate, BHB, ginseng, ginsenosides, healthspan, cognitive function, cardiopulmonary exercise testing, biological age, DNA methylation

## Abstract

**Background:** Nutritional strategies targeting NAD^+^ metabolism, mitochondrial function, and metabolic resilience are gaining attention as potential approaches to healthy aging. This study investigated the short-term physiological, cognitive, and aging-related responses to NMN-based nutritional formulations, including combinations with β-hydroxybutyrate, ginsenosides, and complementary metabolic modulators. **Methods:** In this 42-day, open-label, four-arm exploratory clinical study, 40 Japanese adults aged 45–65 years were included in the final analysis: placebo (*n* = 10), NMN alone (*n* = 9), a core combination (*n* = 10), and an expanded eight-component formulation (*n* = 11). Cardiopulmonary exercise testing (CPET) and computerized cognitive assessments (CogniTrax) were performed at baseline and week 6. Additional exploratory analyses evaluated immune and inflammatory biomarkers and model-derived functional-age estimates in a placebo-versus-expanded-formulation subset. DNA methylation and transcriptomic profiles were independently assessed in a separate eight-participant pilot. **Results:** The intervention was associated with high recorded adherence and no reported serious adverse events. Although between-group comparisons did not demonstrate statistically significant improvements in the selected cardiopulmonary or cognitive outcomes, several cognitive measures showed favorable descriptive changes in the expanded-formulation group relative to placebo, including the Neurocognitive Index (+3.09 vs. −1.90), composite memory (+5.82 vs. −5.30), and verbal memory (+9.00 vs. −2.50). Exploratory biomarker analyses identified nominal between-group differences in CTACK (*p* = 0.015) and GDF-15 (*p* = 0.022), without adjustment for multiple testing; the direction of these changes does not establish clinical benefit. A model-derived functional-age composite decreased by 2.79 years in the expanded-formulation group compared with 0.63 years in placebo. In the separate uncontrolled molecular pilot, DNAm PhenoAge relative to chronological age decreased by approximately 4.1 years, accompanied by nominal transcriptomic changes. These exploratory age-related measures do not establish biological age reversal. **Conclusions:** This exploratory study demonstrates the feasibility of short-term administration of NMN-based multi-ingredient nutritional formulations and provides multidomain descriptive observations across cognitive, physiological, inflammatory, and molecular measures. While the findings do not establish clinical efficacy or ingredient-specific effects, they offer a foundation for hypothesis development and the design of larger, randomized, double-blind trials with prespecified outcomes.

## 1. Introduction

Aging is accompanied by progressive loss of physiological reserve across metabolic, musculoskeletal, cognitive, and immune systems. Geroscience seeks interventions that target mechanisms of aging in order to delay or reduce multiple age-related functional declines rather than treating individual diseases in isolation [1]. Among pathways receiving particular attention are NAD+ metabolism, mitochondrial energetics, nutrient sensing, inflammatory regulation, and cellular stress responses [2,3,4,5,6,7].

Nicotinamide mononucleotide (NMN) is an intermediate in NAD+ biosynthesis. Preclinical studies have linked restoration of NAD+ availability with improved mitochondrial and vascular function, while early human studies indicate that orally administered NMN can increase circulating NAD-related metabolites and has generally been well tolerated [2,3,4,8]. However, clinical evidence for broad functional benefits remains preliminary, and effects may depend on dose, duration, population, and co-interventions.

Beta-hydroxybutyrate (BHB) is both a circulating energy substrate and a signaling metabolite. Ketone signaling has been linked to skeletal-muscle metabolic adaptation, stress resistance, and inflammatory regulation [9,10]. In a randomized trial in Japanese adults, daily D-BHB supplementation was associated with reduction in body fat, supporting the biological activity of exogenous D-BHB in humans [11]. Ginseng-derived ginsenosides have also been investigated for immunomodulatory and neurocognitive effects, with supportive preclinical evidence and small clinical trials of Panax ginseng preparations [12,13,14,15].

We developed several NMN-based drink formulations to examine whether combining these metabolic and nutraceutical components could produce measurable short-term changes across multiple healthspan-relevant domains. Importantly, the most complex formulation evaluated in this study included not only NMN, D-BHB, and a ginseng-derived ingredient but also berberine, pyrroloquinoline quinone (PQQ), coenzyme Q10 (CoQ10), trans-resveratrol, and spermidine. Several of these additional compounds have independent biological plausibility and human data, which makes attribution to any one component impossible in a multi-ingredient trial [16,17,18,19].

The present manuscript reports the completed 42-day, four-arm exploratory clinical study, emphasizing objective CPET and computerized cognitive outcomes. It also reports an exploratory placebo-versus-expanded-formulation analysis of selected immune/inflammatory biomarkers and a BLSA-derived functional-age composite, together with a clearly separated, uncontrolled molecular pilot in eight employees. The primary aim is to characterize feasibility and signal direction rather than to establish definitive efficacy or biological age reversal.

## 2. Materials and Methods

### 2.1. Study Design and Ethical Oversight

We conducted Study 158550-39866 (protocol title: “Verification of Rejuvenation Effects from Food Intake”) in Japan as a 42-day, open-label, four-arm parallel comparative intervention. Participants were allocated with an intention to balance age and sex as practicable. The individuals responsible for assignment and allocation concealment have not been independently documented for the complete four-arm dataset. A separate XPRIZE semifinal recruitment report describes random 1:1 allocation of 22 participants to the placebo and expanded-formulation groups, but it does not establish the randomization method or concealment for the full four-arm population. We therefore do not characterize the entire study as a verified randomized controlled trial.

We conducted the clinical study from 14 October 2025 to 26 December 2025. The protocol was reviewed by the Shiba Palace Clinic Ethics Review Committee on 25 September 2025 and approved on 26 September 2025 (Approval No. 158550-39866). We obtained written informed consent from all participants before participation, and our protocol specified adherence to the Declaration of Helsinki.

**Figure 1:**
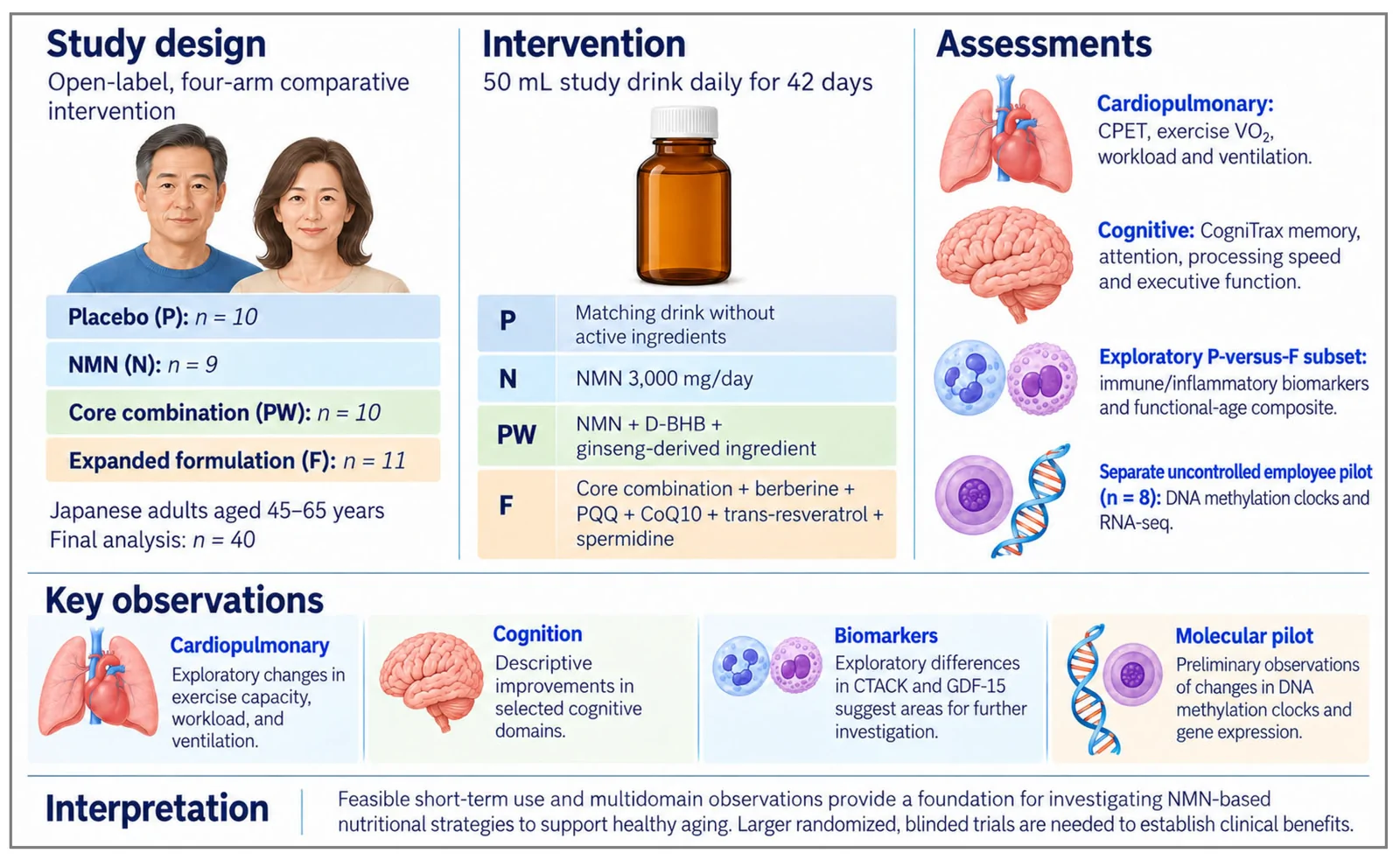
Overview of the 42-day study of NMN-based nutritional formulations in Japanese adults. Summary of the four-arm study design (*n* = 40), interventions, assessments, and key exploratory observations, including a separate uncontrolled employee pilot (*n* = 8). NMN, nicotinamide mononucleotide; CPET, cardiopulmonary exercise testing; VO_2_, oxygen uptake.

The study design, intervention groups, and assessment domains are summarized in Figure 1. Molecular analyses conducted in a separate eight-participant employee pilot are presented independently. 

### 2.2. Participants

Eligible participants were Japanese men and women aged 45–65 years who were considered healthy on the basis of self-report and able to comply with study procedures. Major exclusion criteria included severe gastrointestinal, hepatic, renal, cardiovascular, hematologic, or endocrine disease; suspected or previous malignant neoplasm; diagnosed or suspected major psychiatric disorders such as schizophrenia or severe depression; high risk of harm to self or others; and cognitive impairment that prevented adequate understanding of this study. Participants were instructed to maintain their usual sleep, diet, and general lifestyle throughout this study and to fast for at least 12 h before clinic assessments, with water permitted.

### 2.3. Allocation and Study Products

Participants consumed one 50 mL study drink once daily after breakfast for 42 days. If the morning dose was missed, participants could consume it later the same day but were instructed not to carry a missed dose into the following day. The F formulation was mixed with an equal volume of water before consumption. Study products were supplied by Abe Yoando Pharmaceutical Co., Ltd, Tokyo, Japan. Product labels in the final report were AY-BGN-90000P (P), AY-BGN-90000N (N), AY-BGN-90000PW (PW), and AY-BGN-90000F (F). For scientific clarity, we refer to these as placebo, NMN-only, core combination, and expanded combination, respectively. The sponsor developed, manufactured, and supplied the formulations; SOUKEN Co., Ltd, Tokyo, Japan managed trial operations and prepared the clinical study report. Ingredient quantities reported for AY-BGN-90000F in a subsequent April 2026 patent-related formulation document differ from the trial formulation in Table 1 (notably D-BHB, the ginseng preparation, and resveratrol). The table retains the composition in the supplied final clinical study report pending batch-specific verification.

### 2.4. Cardiopulmonary Exercise Testing

CPET was performed using a cycle ergometer under standardized pre- and post-intervention conditions. Participants rested for 3 min, completed a 3 min warm-up at 0 W, and then underwent a progressive load increase of approximately 10–25 W/min according to individual fitness, with an exercise period of approximately 10 min terminated at perceived maximal intensity. Recorded variables included resting and exercise oxygen consumption (VO2), carbon dioxide production (VCO2), respiratory exchange ratio (RER), minute ventilation (VE), maximal ventilation (VEmax), anaerobic threshold (AT), and achieved workload. The source documents do not establish the ergometer model, metabolic cart model, calibration schedule, objective maximal-effort thresholds (including VO2 plateau or RER/heart-rate criteria), AT determination algorithm, or assessor/equipment consistency. Accordingly, observed oxygen uptake is reported as exercise VO2 rather than verified VO_2_max.

### 2.5. Cognitive Assessment

Cognitive function was assessed with CogniTrax, a computerized battery standardized for use in Japan. The final report included the Neurocognitive Index (NCI) and domain scores for composite memory, verbal memory, visual memory, cognitive processing speed, reaction time, complex attention, cognitive flexibility, processing speed, executive function, simple attention, and motor speed. Standardized scores were emphasized in the present manuscript because they facilitate comparison across cognitive domains. The original documentation does not establish whether alternate CogniTrax forms or a familiarization session were used at follow-up. Practice, expectancy, and repeated-testing effects may therefore influence observed changes.

### 2.6. Immune and Inflammatory Biomarker Subset

A competition-focused exploratory subset compared the placebo arm with the expanded-combination arm. Serum was assayed using the MILLIPLEX Human Aging Magnetic Bead Panel 1 (Millipore, Billerica, MA, USA; HAGE1MAG-20K-10) on a Bio-Plex 200 system with Bio-Plex Manager Software Version 6.1. Measured analytes included CTACK/CCL27, FGF-21, GDF-11, GDF-15, GnRH, IL-6, IL-10, IL-18, and leptin. Several values were below the assay detection limit in these healthy participants; therefore, biomarker analyses were exploratory, and analyte-specific sample availability varied.

### 2.7. Adherence and Safety Monitoring

Study-product use was recorded during the 42-day intervention. The supplied adherence dataset indicates 42/42 scheduled doses for each of the 40 participants in the final four-arm analysis population; this is recorded adherence, not necessarily independently verified ingestion. The XPRIZE semifinal subset also reports full reported adherence and no serious adverse events. The method used to verify each dose (diary, bottle count, interview, or other measure) was not established by the available source documentation. Participants were asked to report symptoms during dosing and for two weeks afterward. According to SOUKEN Co., Ltd.’s study records, recorded adherence was 100% among the 40 analyzed participants, with no serious adverse events reported.

### 2.8. Exploratory Functional-Age Analysis

For the placebo-versus-expanded-formulation subset, an exploratory functional-age analysis converted changes in selected physiological measures into age-equivalent changes using fixed age slopes derived from the Baltimore Longitudinal Study of Aging (BLSA) framework [20,21]. The de-identified table included 11 measures spanning CPET (exercise VO2, workload, and AT), cognition (visual memory, cognitive processing speed, executive function, and complex attention), and serum biomarkers (GDF-15, CTACK, IL-6, and IL-10). Metric-specific changes were converted to age-gap values using the stated aging slopes and integrated into an estimated functional-age change. This composite has not been established as a validated surrogate endpoint for clinical benefit and was treated as exploratory. The converted age-equivalent units constitute a mathematical transformation, not a validated estimate of a change in chronological or biological age and not a surrogate for clinical benefit.

### 2.9. Independent Exploratory Molecular Pilot

A separate convenience sample of eight employees aged 40–54 years received the candidate supplement for 42 days. This uncontrolled cohort was independent of the four-arm clinical study, and prior NMN use was noted as possible. Blood was sampled before and after intake; DNA methylation profiling was performed by Rhelixa Inc, Tokyo, Japan. using Illumina iScan and SeSAMe preprocessing. Multiple epigenetic clocks were evaluated [22,23,24], but prospective selection of DNAm PhenoAge as the principal endpoint was not documented. This pilot is presented as methodological context only; its full descriptive results and limitations belong in Supplementary Materials and cannot support clinical efficacy or age-reversal claims.

RNA-seq in the separate employee pilot used TruSeq Stranded Total RNA (Illumina, Inc., San Diego, CA, USA) with Ribo-Zero Globin, GRCh38/hg38 alignment, and NovaSeq X Plus 150-bp paired-end sequencing. The source report gives approximately 12 Gb and 80 million reads per sample and nominal differential-expression findings; an across-gene false-discovery-rate threshold was not documented. Two post-intervention samples were excluded from a selected-gene heatmap for quality concerns. Gene and pathway observations are descriptive, sensitive to multiple testing, and detailed in Supplementary Materials rather than interpreted as established molecular effects.

### 2.10. Statistical Analysis

For the four-arm clinical dataset, change scores were defined as week-6 minus baseline and summarized using mean and standard deviation. The available final statistical workbook reports pairwise Tukey comparisons, but the parent model (e.g., one-way ANOVA on change scores), model assumptions, and use of baseline adjustment were not documented in the materials available to the authors. Therefore, the existing reported comparisons are reproduced descriptively and cannot be interpreted as confirmatory. No single primary efficacy endpoint or full-endpoint multiplicity plan was clearly documented.

The separate semifinal placebo-versus-expanded-formulation analysis compared pre-to-post change scores using two-sided Mann–Whitney U tests, as documented in the semifinal technical application; CTACK and GDF-15 were re-evaluated from available de-identified participant change scores. This distinct two-arm analysis must not be conflated with the four-arm Tukey comparisons. No across-analyte false-discovery or familywise multiplicity adjustment was documented. All reported significance levels are nominal exploratory observations.

## 3. Results

### 3.1. Participant Disposition and Baseline Characteristics

Forty-two participants initiated this clinical study. Two participants withdrew for personal reasons, after which we analyzed 40 participants: P (*n* = 10), N (*n* = 9), PW (*n* = 10), and F (*n* = 11). In our separate semifinal P-vs-F analysis, all 11 participants in F were included, while 10 control participants had evaluable data; the semifinal recruitment documentation attributed exclusion of one control dataset to a technical interruption during computerized cognitive testing. The semifinal recruitment report separately documents 22 participants randomized as two groups (11/11), with 21 analyzed after exclusion of one control dataset because of a computerized test interruption. These are separate reporting/analysis populations and do not imply that the 22-person semifinal sample independently establishes participant flow or randomization across the complete 42-person four-arm cohort. The participant populations and analyses are reported separately to ensure transparency (Table 2).

The F group was older on average than the P group (60.8 vs. 55.8 years), an imbalance that is relevant to interpretation of age-sensitive physiological and cognitive endpoints.

**Figure 2:**
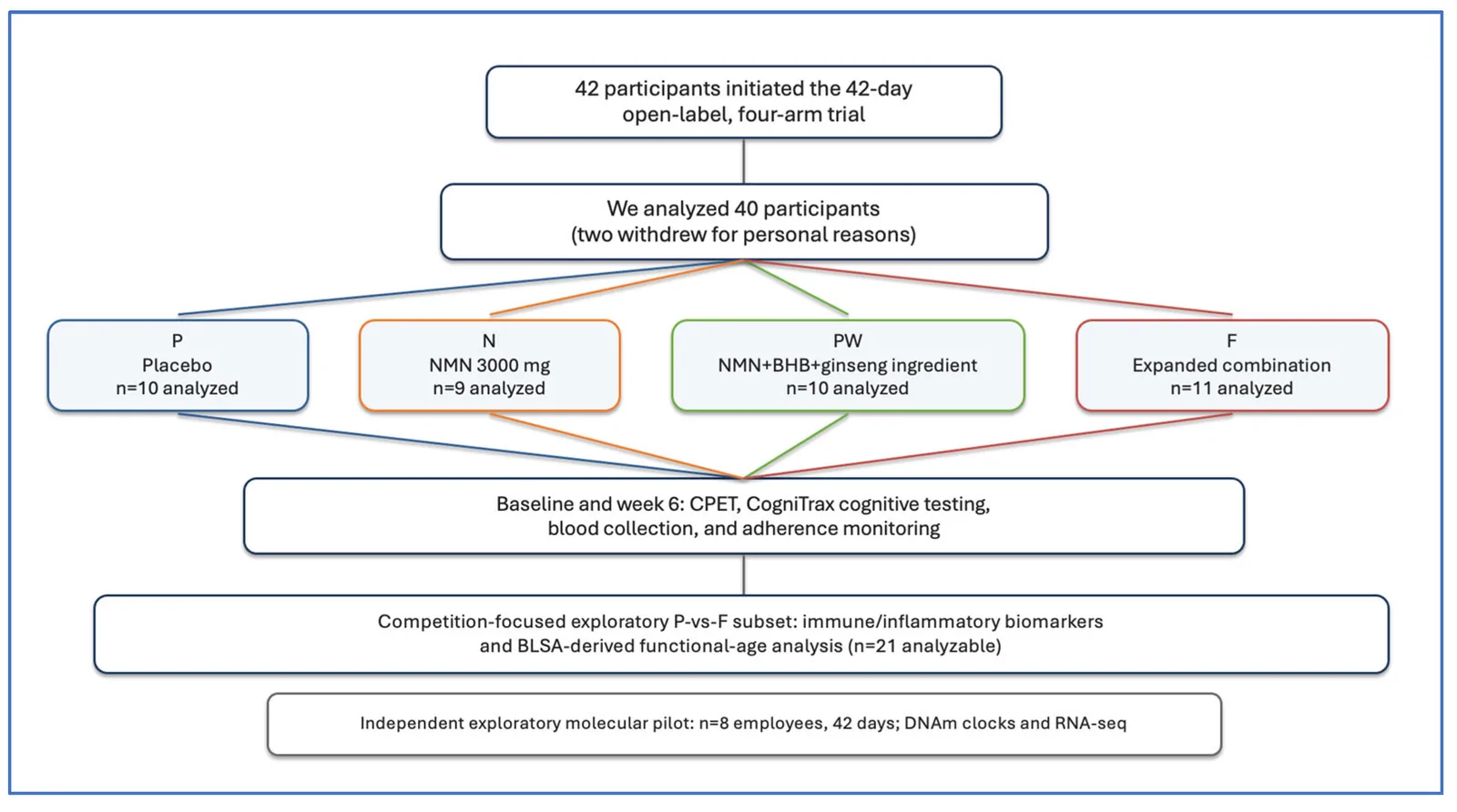
Study design and analysis populations reconstructed from the formal final report and supplied semifinal datasets. The independent molecular pilot was a separate uncontrolled cohort.

Study design and analysis populations. Overview of the 42-day, open-label, four-arm exploratory clinical study and associated analysis populations. Participants received placebo (P), NMN alone (N), NMN + D-beta-hydroxybutyrate (D-BHB) + ginseng-derived ingredient (PW), or the expanded multi-ingredient formulation (F). Forty-two participants initiated this study, and 40 were included in the final four-arm analysis. The placebo-versus-expanded-formulation subset was used for exploratory immune/inflammatory biomarker and BLSA-derived functional-age analyses. The independent 8-participant molecular pilot was a separate, uncontrolled cohort and was not part of the four-arm clinical study (Figure 2).

### 3.2. Adherence and Available Safety Information

The supplied adherence workbook records 42 of 42 scheduled doses for each of the 40 participants retained in the four-arm analysis; these should be referred to as recorded, not objectively verified, adherence until the underlying method is confirmed. The separate semifinal report documents no serious adverse events or unexpected problems; no evidence in the supplied materials attributes the two four-arm withdrawals to treatment. 

### 3.3. Cardiopulmonary Exercise Outcomes

Exercise CPET change scores did not show statistically significant pairwise differences between P and F for AT, workload, exercise VO2, VCO2, RER, VE, or VEmax. Selected change scores are summarized in Table 3. The direction of change was mixed rather than consistently favoring the expanded combination. For example, exercise workload changed by −5.71 ± 15.23 W in P and −1.19 ± 11.79 W in F (P-vs-F Tukey *p* = 0.951), while exercise VO2 changed by +0.0018 ± 0.0846 L/min in P and −0.0585 ± 0.1013 L/min in F (*p* = 0.624).

At rest, most variables also showed no between-group differences. One isolated comparison reached nominal significance: resting VEmax change differed between P (−0.085 ± 0.150 L/min) and N (+0.115 ± 0.166 L/min; Tukey *p* = 0.039). Given the large number of outcomes, this isolated nominal result should not be regarded as confirmatory.

### 3.4. Cognitive Outcomes

Several standardized cognitive domains showed positive mean changes in N, PW, or F, while P showed small negative mean changes. In F compared with P, the NCI changed by +3.09 ± 10.14 versus −1.90 ± 8.67, composite memory by +5.82 ± 13.62 versus −5.30 ± 20.54, verbal memory by +9.00 ± 24.41 versus −2.50 ± 22.90, cognitive processing speed by +5.91± 25.07 versus −2.10 ± 15.69, and motor speed by +7.55 ± 34.43 versus −2.40 ± 14.47. However, none of the P-vs-F contrasts in the standardized domain scores reached statistical significance. The N arm showed a comparatively large mean NCI increase (+13.11 ± 17.78), but the P-vs-N comparison was also not statistically significant at the 0.05 level (*p* = 0.062) (Table 4).

**Figure 3:**
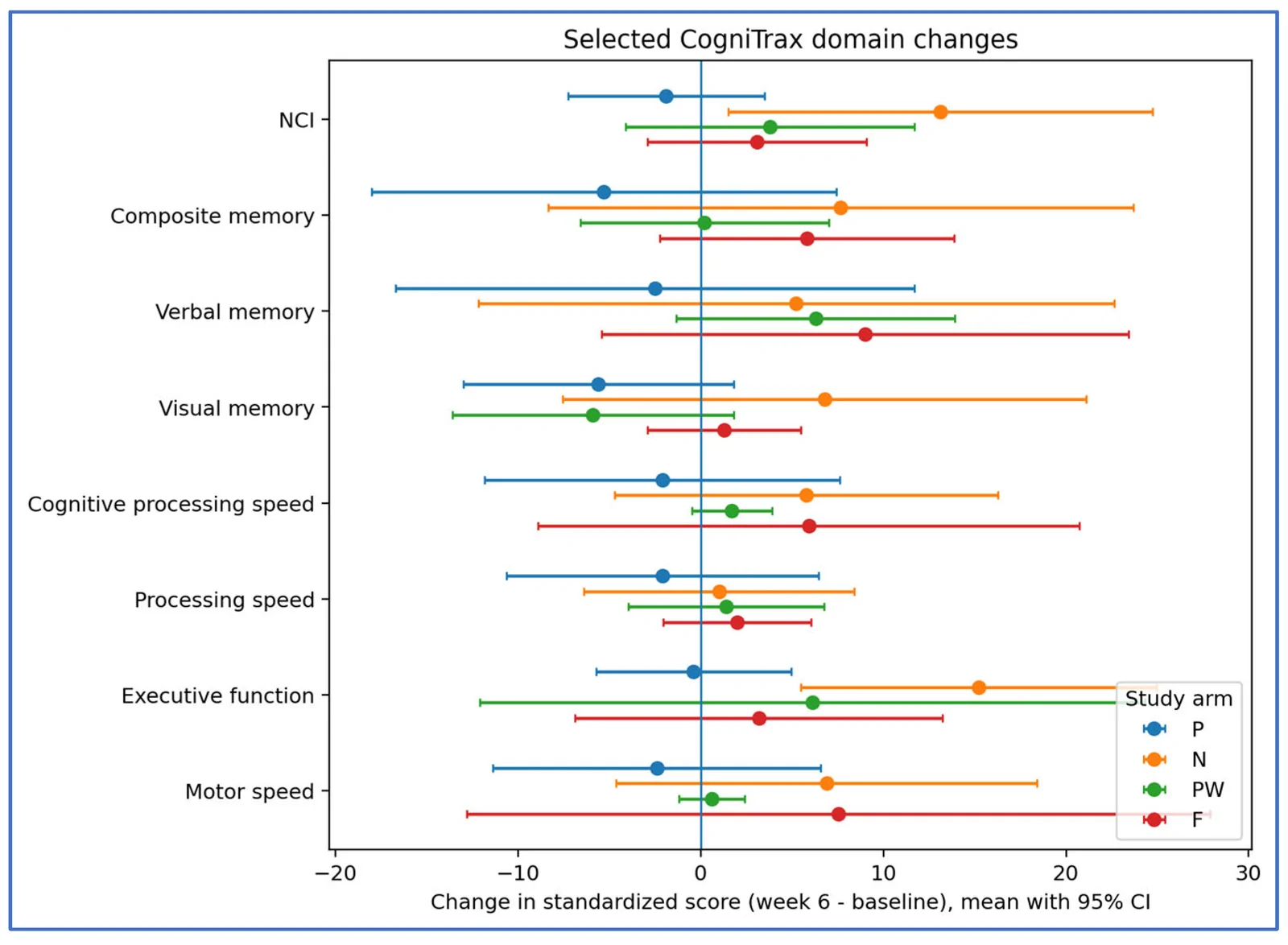
Mean change and approximate 95% confidence intervals for selected standardized CogniTrax scores across the four study arms. Confidence intervals are descriptive and calculated from the supplied mean, SD, and n; formal pairwise testing in the source workbook used Tukey tests.

Figure 3 changes in selected standardized CogniTrax cognitive scores. Mean change from baseline to week 6 with approximate 95% confidence intervals for selected standardized CogniTrax cognitive domains across the placebo (P), NMN-only (N), core-combination (PW), and expanded-combination (F) groups. Confidence intervals are descriptive and were calculated from the reported mean, standard deviation, and sample size. Formal pairwise comparisons in the source statistical workbook used Tukey tests. Positive change generally indicates improved performance for the standardized scores shown.

### 3.5. Exploratory Immune and Inflammatory Biomarkers

Immune biomarker analysis was available for the placebo-versus-expanded-formulation subset rather than the complete four-arm cohort. De-identified raw data showed substantial inter-individual variability and frequent values below the limit of detection for several cytokines. For analytes with complete paired values, the mean CTACK change was −441.6 pg/mL in P and +203.0 pg/mL in F; a two-sided Mann–Whitney U reanalysis of individual change scores yielded *p* = 0.015. GDF-15 changed by −188.6 pg/mL in P and +109.3 pg/mL in F (*p* = 0.022) (Table 5).

CTACK and GDF-15 differences were nominal results of unadjusted tests in a small exploratory subset and should not be interpreted as evidence of healthspan benefit. The expanded-formulation arm showed an increase in GDF-15, a context-dependent stress marker; neither the direction nor the clinical implications establish a favorable intervention effect. Several other analytes were below the detection limit, further limiting interpretation.

### 3.6. Exploratory Functional-Age Analysis

Using the BLSA-derived fixed-slope transformation, the supplied semifinal analysis reported a mean estimated functional-age change of −2.79 years in F compared with −0.63 years in P over 42 days. The proportion classified as responders was 72.7% in F and 50.0% in P. Because the composite was constructed from multiple study outcomes, used model-derived aging slopes, and has not been validated as a surrogate endpoint for clinical outcomes in this setting, these values should be interpreted as a descriptive composite signal rather than literal reversal of chronological or biological aging.

**Figure 4:**
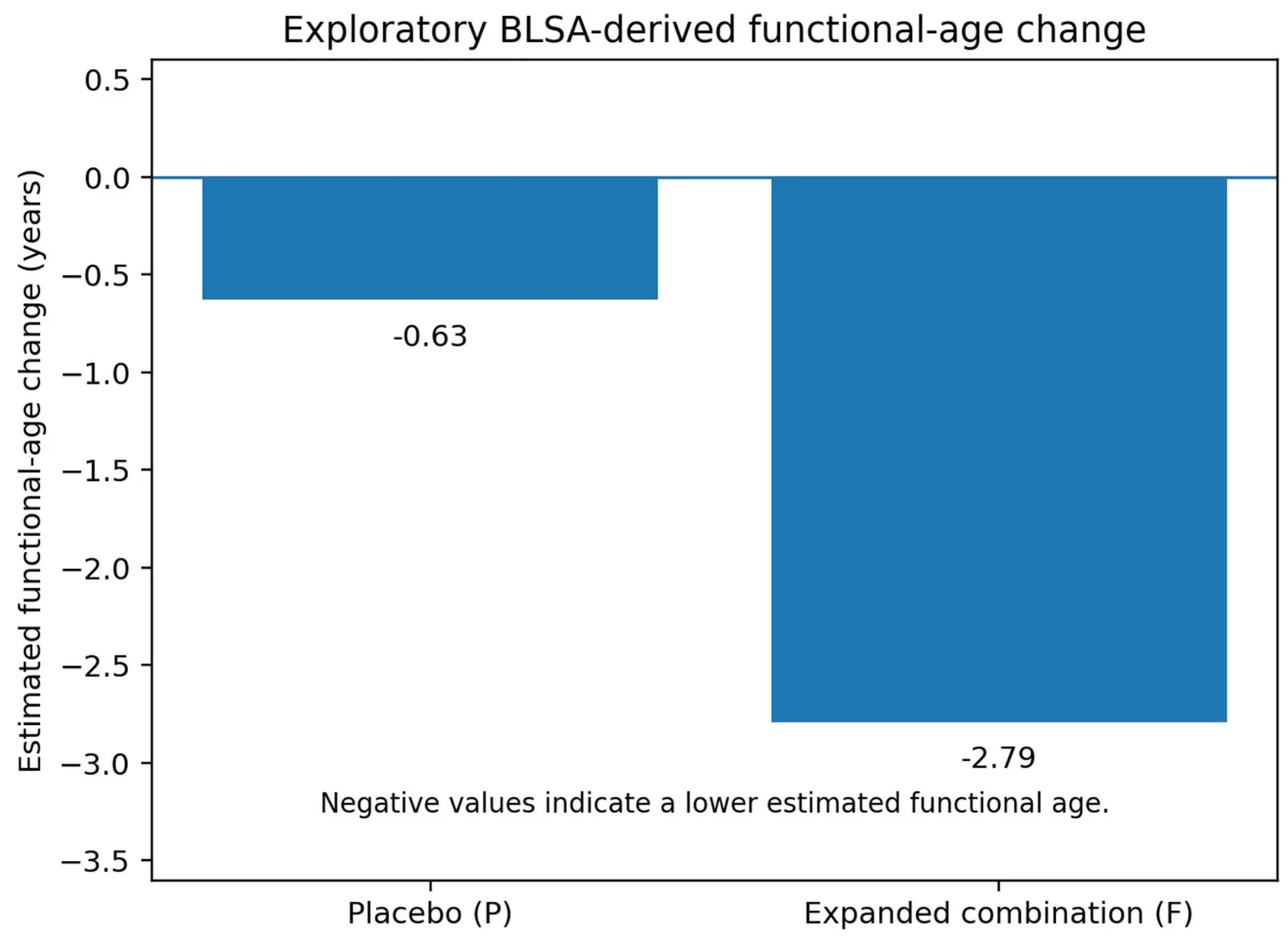
Exploratory mean BLSA-derived functional-age change in the placebo and expanded-combination groups. Negative values indicate lower estimated functional age. No causal age-reversal interpretation is warranted.

Exploratory BLSA-derived functional-age change. Mean estimated functional-age change over 42 days in the placebo (P) and expanded-combination (F) groups using a Baltimore Longitudinal Study of Aging (BLSA)-derived fixed-slope transformation. Mean functional-age change was −0.63 years in P and −2.79 years in F. Negative values indicate lower estimated functional age. This exploratory composite has not been validated as a surrogate endpoint for clinical benefit and should not be interpreted as evidence of biological or chronological age reversal (Figure 4).

### 3.7. Independent Molecular Pilot

The separate, uncontrolled eight-person employee pilot is described in Supplementary Methods and Results. It examined several DNA methylation clocks and RNA-seq outcomes after 42 days without a concurrent control group. Because clock choice was not documented as prespecified, baseline values were already younger than chronological age, prior supplement use was possible, and multiple-testing control was unavailable for RNA-seq, the pilot cannot be used to infer intervention efficacy or age reversal.

RNA-seq analysis identified 121 nominally upregulated and 122 nominally downregulated genes. Reported examples included increases in EGR3, FOSB, MYOZ1, AK1, LINGO4, NEAT1, and CCL3L3 and decreases in IFI27, PTGES, SPP1, GLUD2, and CPNE4. KEGG gene-set analyses highlighted oxidative phosphorylation and thermogenesis, actin-cytoskeleton regulation, synaptic/neurotrophin-related pathways, and B-cell, T-cell, cytokine, and interferon-related immune pathways. These analyses did not include a concurrent control group, and the supplied summary did not document false-discovery-rate control for the differential-expression screen; therefore, mechanistic interpretations remain preliminary.

### 3.8. Principal Findings

This study primarily documents feasibility and descriptive 42-day outcomes of four nutritional formulations. The selected placebo-versus-expanded-formulation CPET and standardized cognitive comparisons were not statistically significant. Differences in mean change directions do not demonstrate treatment effects and could reflect baseline imbalance, expectancy, testing familiarity, or chance. Nominal immune-marker observations were unadjusted and biologically uncertain. An uncontrolled molecular pilot is reported separately for hypothesis generation, not as supporting clinical efficacy.

### 3.9. Interpretation of Cognitive and Physical Findings

Cognitive aging reflects changes in memory, processing speed, executive control, and attention, domains that can also be influenced by learning effects, sleep, motivation, and repeated testing. The favorable mean direction seen in several CogniTrax standardized domains is therefore encouraging but not definitive. Practice effects are especially important in a short repeated-measures design, and the open-label nature of this study may further influence performance. The absence of statistically significant P-vs-F differences across the domain battery argues against strong efficacy claims from this sample.

Similarly, the CPET results did not demonstrate a consistent treatment signal across objective exercise variables. The isolated nominal difference in resting VEmax between P and N should be interpreted cautiously because many physiological outcomes were tested. A larger trial should prespecify a small number of primary physical endpoints, use blinded assessment, and employ models that adjust for baseline values and relevant covariates rather than relying predominantly on multiple change-score comparisons.

### 3.10. Immune and Inflammatory Signals

The small placebo-versus-expanded-formulation biomarker subset yielded nominal, unadjusted differences in CTACK and GDF-15 change scores. CTACK relates to cutaneous immune trafficking and is not a validated healthspan surrogate. GDF-15 increased in the expanded-formulation arm and may reflect physiological stress in some contexts [25]. The frequency of measurements below detection limits and the number of analytes examined preclude conclusions about beneficial immunomodulation or treatment-associated healthspan effects.

### 3.11. Biological Plausibility and Formulation Complexity

Interpretability is fundamentally constrained by the eight-active-ingredient expanded formulation, including NMN, D-BHB, a ginseng-derived preparation, berberine, PQQ, CoQ10, resveratrol, and spermidine. The design does not isolate any component, permit claims of synergy, or establish a coherent treatment mechanism. Hypothesized NAD-related, metabolic, or immunological pathways must remain background rationale, not demonstrated biological effects; component-deconstruction or factorial trials would be needed for attribution.

### 3.12. Functional Age and Epigenetic Age

Composite or molecular age estimates can be useful for integrating multidimensional information, but their interpretation requires caution. The BLSA-derived functional-age analysis transformed short-term changes in study variables into age-equivalent units using fixed aging slopes [20,21]. This creates an intuitive summary but does not mean that 42 days of treatment changed an individual’s chronological age or established a validated reduction in morbidity risk. Because several components of the composite were themselves study outcomes, the functional-age estimate is not independent confirmation of the component results.

In the separate eight-person uncontrolled employee pilot, DNA methylation age estimates were already younger than chronological age before intervention, some participants may previously have used NMN, and DNAm PhenoAge was not documented as prospectively prespecified among several clocks. Quality exclusions and absent transcriptome-wide FDR documentation further limit reliability. The reported short-term difference in a clock estimate does not constitute evidence of reversal of biological aging. Detailed observations are presented only as supplementary, hypothesis-generating data.

### 3.13. Strengths

Strengths include a prospective 42-day intervention period, objective CPET testing, a standardized computerized cognitive battery, participant-level adherence data, a placebo-containing study arm, collection of de-identified individual biomarker data, and an attempt to examine multiple physiological domains relevant to healthspan. The availability of NMN-only, core-combination, and expanded-combination arms also provides useful information for planning future component-focused research, even though the present sample was too small for reliable between-formulation comparisons.

### 3.14. Limitations

This study was small and open-label, with no clearly documented concealed allocation for the entire four-arm population, a five-year mean age imbalance between placebo and the expanded-formulation arm, no prespecified primary efficacy endpoint, and no multiplicity plan covering the many clinical and biomarker outcomes. The underlying model for reported four-arm Tukey comparisons and the verification of complete adherence require clarification from the CRO. Test familiarity and expectancy could affect CogniTrax scores, while CPET equipment/calibration and maximal-effort criteria were incompletely documented. The eight-component formulation prevents ingredient-specific or synergistic attribution. Exploratory biomarker and functional-age measures lack validated clinical surrogacy, and the distinct uncontrolled molecular pilot is subject to selection bias, prior exposure, clock-selection uncertainty, and unadjusted transcriptomic multiple testing. These constraints prohibit causal efficacy and age-reversal interpretations.

### 3.15. Implications for a Confirmatory Trial

The data support proceeding, if desired, to a rigorously designed confirmatory study rather than making strong claims from the present exploratory dataset. A future trial should be prospectively registered before enrollment, randomized with concealed allocation, double-blind, and placebo controlled. A single primary endpoint or a small hierarchical set of endpoints should be specified in advance. Baseline-adjusted linear models or mixed-effects models should be used, with explicit multiplicity control for secondary domains. The intervention should also be chemically standardized, including exact active ginsenoside content and stability testing. If mechanistic synergy is a central claim, a factorial or component-deconstruction design is necessary. Epigenetic and transcriptomic outcomes should be prespecified, processed in randomized laboratory batches, analyzed with false-discovery-rate control, and interpreted independently of clinical outcomes.

## 4. Conclusions

In this 42-day open-label, four-arm exploratory study, the selected placebo-versus-expanded-formulation cardiopulmonary and cognitive comparisons were not statistically significant. Recorded dosing completion and feasibility observations warrant appropriately controlled follow-up. Nominal biomarker differences, mathematically derived age-equivalent scores, and the separate uncontrolled molecular pilot do not establish improvement in healthspan, effects of individual ingredients, synergy, or biological age reversal. Future trials require documented concealed randomization, blinded assessment, prespecified primary endpoints, appropriate multiplicity control, verified formulation specifications, and longer safety follow-up.

These findings provide a foundation for larger, randomized, double-blind trials to further investigate the potential of NMN, both alone and in combination with complementary bioactive ingredients, to support healthy aging and physiological resilience.

## Figures and Tables

**Table 1:** Study formulations, transcribed from the final clinical study report. Quantities and active ginsenoside standardization require reconciliation against the actual 2025 administered-batch manufacturing records before publication; the later patent-development composition must not be substituted without verification.

Arm	Composition per Daily Bottle (as Listed in Final Report)	Energy/ Carbohydrate
P: placebo	Glucose syrup (~7%); herbal flavor; water to 50 mL	14 kcal; 3.5 g carbohydrate
N: NMN only	beta-NMN 3000 mg; glucose syrup; flavor/color; water to 50 mL	14 kcal; 3.5 g carbohydrate
PW: core combination	beta-NMN 3000 mg; D-BHB 200 mg; RED JINSEN K(R) ginseng-derived ingredient 300 mg; glucose syrup; yuzu flavor; water to 50 mL	15 kcal; 3.5 g carbohydrate
F: expanded combination	PW core ingredients plus berberine 50 mg, PQQ 21.5 mg, CoQ10 200 mg, trans-resveratrol 21.5 mg, and spermidine 2.0 mg	15 kcal; 3.5 g carbohydrate

**Table 2:** Baseline characteristics of the final four-arm analysis population. Values are mean **±** SD unless otherwise stated.

Characteristic	P	N	PW	F
Analyzed, n	10	9	10	11
Age, years	55.8 ± 4.9	56.6 ± 7.0	56.7 ± 5.7	60.8 ± 5.2
Male, n	2	1	2	3
Female, n	8	8	8	8

**Table 3:** Change from baseline to week 6 in selected exercise CPET variables. Values are mean +/− SD; *p* values are pairwise Tukey comparisons reported in the final statistical workbook.

Exercise CPET Change	P	N	PW	F	P vs. F *p*
AT, W	+3.00 ± 13.78	−5.00 ± 22.50	−3.00 ± 31.46	+1.36 ± 17.04	0.998
Workload, W	−5.71 ± 15.23	−5.09 ± 15.58	+1.84 ± 30.46	−1.19 ± 11.79	0.951
VO2, L/min	+0.0018 ± 0.0846	−0.0193 ± 0.1333	−0.0088 ± 0.1332	−0.0585 ± 0.1013	0.624
VCO2, L/min	−0.0025 ± 0.1010	−0.0333 ± 0.1586	+0.0538 ± 0.1682	−0.0262 ± 0.1262	0.980
RER	+0.0073 ± 0.0548	−0.0149 ± 0.0497	+0.0576 ± 0.0946	+0.0343 ± 0.0487	0.776
VE, L/min	−0.0007 ± 0.1781	−0.0525 ± 0.2894	+0.0450 ± 0.2933	−0.0847 ± 0.2230	0.866
VEmax, L/min	−0.218 ± 0.287	−0.498 ± 0.675	−0.398 ± 0.645	−0.445± 0.344	0.739

**Table 4:** Change in standardized CogniTrax domain scores. Values are mean +/− SD; *p* values are P-vs-F Tukey comparisons. Positive change generally denotes better performance for these standardized scores, but domain-specific interpretation should follow CogniTrax conventions.

Standardized Score Change	P	N	PW	F	P vs. F *p*
Neurocognitive Index	−1.90 ± 8.67	+13.11 ± 17.78	+3.80 ± 12.75	+3.09 ± 10.14	0.801
Composite memory	−5.30 ± 20.54	+7.67 ± 24.51	+0.20 ± 10.97	+5.82 ± 13.62	0.495
Verbal memory	−2.50 ± 22.90	+5.22 ± 26.64	+6.30 ±12.30	+9.00 ± 24.41	0.639
Visual memory	−5.60 ± 11.93	+6.78 ± 21.95	−5.90 ± 12.44	+1.27± 7.09	0.676
Cognitive processing speed	−2.10 ± 15.69	+5.78 ± 16.08	+1.70 ± 3.53	+5.91 ± 25.07	0.713
Complex attention	−0.60 ± 13.13	+27.22 ± 32.67	+9.30 ± 37.84	+2.09 ± 17.48	0.996
Cognitive flexibility	+0.10 ± 9.79	+21.11 ± 23.14	+6.00 ± 29.44	+3.27 ± 16.92	0.985
Processing speed	−2.10 ± 13.79	+1.00 ± 11.31	+1.40 ± 8.66	+2.00 ± 6.84	0.803
Executive function	−0.40 ± 8.62	+15.22 ± 14.94	+6.10 ± 29.39	+3.18 ± 17.03	0.973
Simple attention	−0.80 ± 11.63	+11.78 ± 38.75	−2.00 ± 13.95	−6.09 ± 14.47	0.944
Motor speed	−2.40 ± 14.47	+6.89 ± 17.63	+0.60 ± 2.91	+7.55 ± 34.43	0.710

**Table 5:** Exploratory immune/inflammatory biomarker results in the P-vs-F subset. CTACK and GDF-15 *p* values were recalculated from the supplied de-identified change scores using Mann–Whitney U tests; no across-biomarker multiplicity correction was applied.

Biomarker	P Mean Change	F Mean Change	Two-Sided *p*	Comment
CTACK/CCL27 (pg/mL)	−441.6	+203.0	0.015	Complete paired raw values; exploratory
GDF-15 (pg/mL)	−188.6	+109.3	0.022	Complete paired raw values; exploratory
Other panel analytes	FGF-21, GDF-11, GnRH, IL-6, IL-10, IL-18, leptin	Varied	Not emphasized	Several measurements below detection limit

## Data Availability

De-identified participant-level data were available to the authors for the analyses reported here.

## Supplementary Materials

The following supporting information can be downloaded at https://www.mdpi.com/article/10.3390/nu18213490/s1: Supplementary Table S1. CPET results at baseline, week 6, and change scores; Supplementary Table S2. CogniTrax domain and index results; Supplementary Table S3. Participant-level de-identified immune biomarker data with detection-limit flags; Supplementary Table S4. BLSA functional-age transformation slopes and participant-level derived values; Supplementary Figure S1. RNA-seq volcano plot from the independent molecular pilot; Supplementary Figure S2. KEGG gene-set enrichment results; Supplementary Figure S3. Selected-gene heatmap, with quality-control exclusions documented; Supplementary File S1. Mean DNAmPhenoAge-minus-chronological-age values in the independent 8-participant employee pilot. The cohort was uncontrolled and separate from the clinical trial; uncertainty intervals were not available in the supplied summary.
