# Supplementary material for: Unlocking the Healthy Aging Potential of Novel NMN-Based Nutritional Formulations: Cardiopulmonary, Cognitive, and Aging-Related Outcomes in a 42-Day Open-Label Study
Source: Nutrients. 2026 Oct 23;18(21):3490. doi: 10.3390/nu18213490 (PMC13765308; doi:10.3390/nu18213490)
Supplement: Supplementary file 1 [file nutrients-18-03490-s001.zip › Supplementary Figure S1.pdf]

Supplementary Figure S1. RNA-seq volcano plot from the independent molecular pilot

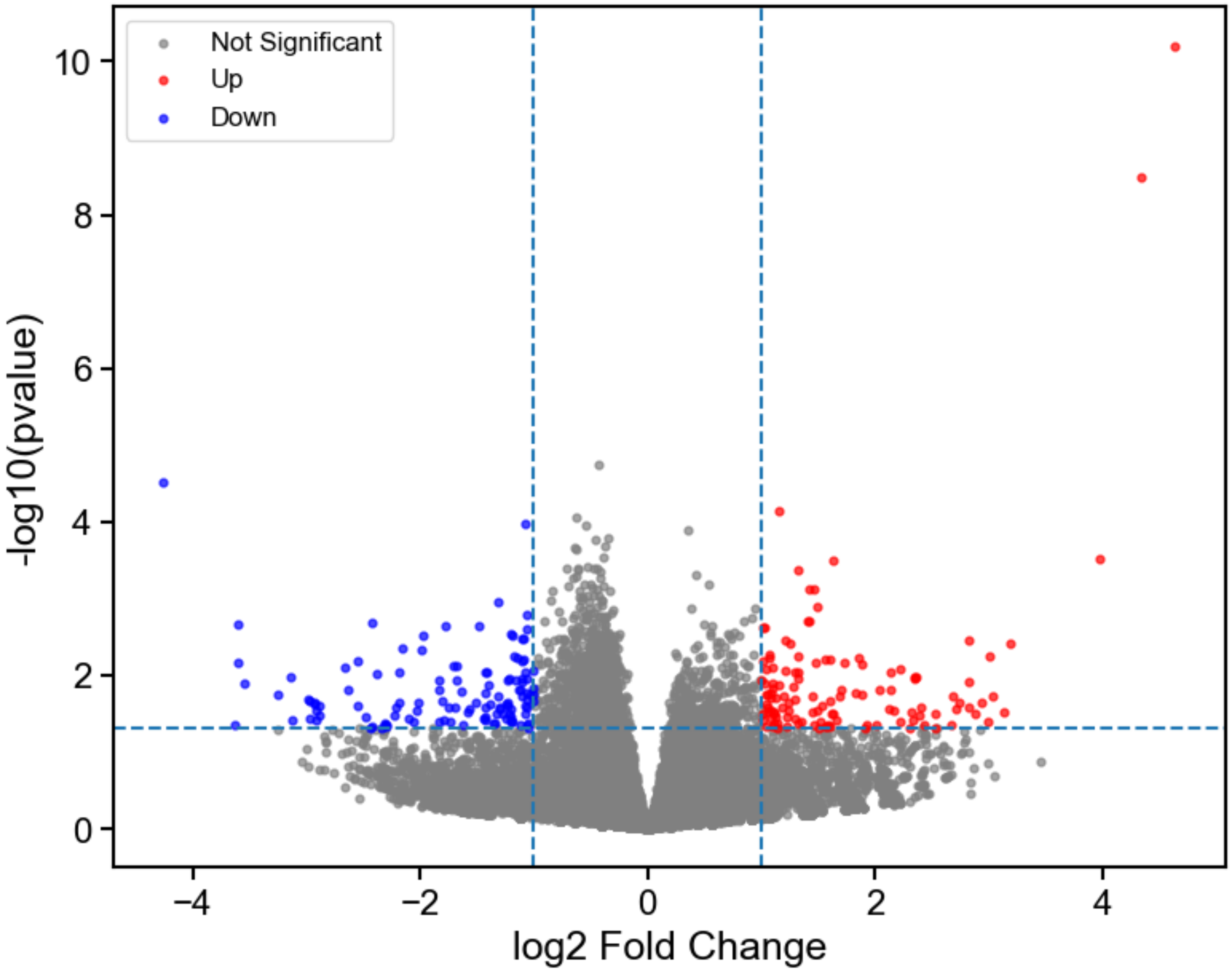

Volcano plot of RNA-seq expression differences between pre- and post-intervention samples from the independent employee molecular pilot. The x-axis shows log2 fold change and the y-axis shows -log10(unadjusted p-value). Red and blue points indicate up- and downregulated genes, respectively, using the source thresholds $p < 0.05$ and |log2 fold change| > 1; grey points fall outside these thresholds.

[illegible]
