# Supplementary material for: Unlocking the Healthy Aging Potential of Novel NMN-Based Nutritional Formulations: Cardiopulmonary, Cognitive, and Aging-Related Outcomes in a 42-Day Open-Label Study
Source: Nutrients. 2026 Oct 23;18(21):3490. doi: 10.3390/nu18213490 (PMC13765308; doi:10.3390/nu18213490)
Supplement: Supplementary file 1 [file nutrients-18-03490-s001.zip › Supplementary_Figure_S3.pdf]

Supplementary Figure S3. Selected-gene heatmap, with quality-control exclusions documented

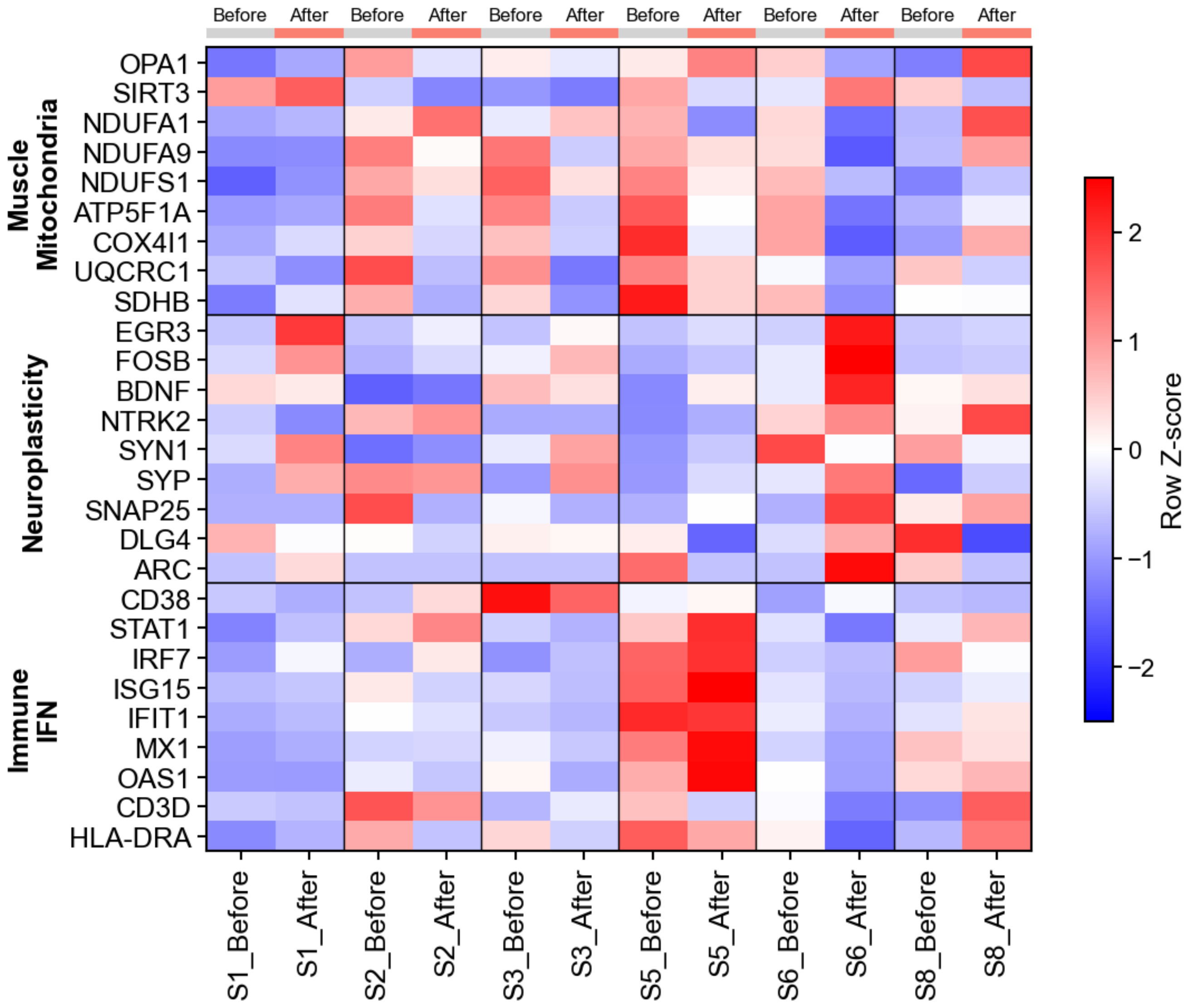

Heatmap of 27 selected genes related to mitochondrial function, neuroplasticity and immune/interferon responses in the independent employee molecular pilot.
