# Supplementary material for: Unlocking the Healthy Aging Potential of Novel NMN-Based Nutritional Formulations: Cardiopulmonary, Cognitive, and Aging-Related Outcomes in a 42-Day Open-Label Study
Source: Nutrients. 2026 Oct 23;18(21):3490. doi: 10.3390/nu18213490 (PMC13765308; doi:10.3390/nu18213490)
Supplement: Supplementary file 1 [file nutrients-18-03490-s001.zip › Supplementary_File_S1.pdf]

Supplementary File S1. Mean DNAmPhenoAge-minus-chronological-age values in the independent 8-participant employee pilot

This uncontrolled employee cohort was independent of, and separate from, the clinical trial. The values below reproduce the approximate means reported in the supplied preliminary summary.

Measurement	N	Mean age gap (years)
Before intervention	8	Approximately -6.9
After intervention	8	Approximately -11.0
Change in reported means	8	Approximately -4.1

Age gap = DNAmPhenoAge minus chronological age. Negative values indicate that the estimated methylation age is below the recorded chronological age. The change shown is the difference between the approximate reported means.
