# Supplementary material for: Unlocking the Healthy Aging Potential of Novel NMN-Based Nutritional Formulations: Cardiopulmonary, Cognitive, and Aging-Related Outcomes in a 42-Day Open-Label Study
Source: Nutrients. 2026 Oct 23;18(21):3490. doi: 10.3390/nu18213490 (PMC13765308; doi:10.3390/nu18213490)
Supplement: Supplementary file 1 [file nutrients-18-03490-s001.zip › Supplementary_Table_S1.pdf]

Supplementary Table S1. CPET results at baseline, week 6, and change scores

Values are mean (sample SD), calculated from the supplied participant records. Change = week 6 minus baseline. CTRL and INTV retain the source group definitions. Before/After source measurements are presented as baseline/week 6 according to the reported 42-day clinical protocol.

Outcome	Unit	Group	N	Baseline	Week 6	Change
VO2 rest	L/min	CTRL	10	0.198 (0.053)	0.205 (0.050)	0.007 (0.019)
VO2 rest	L/min	INTV	11	0.187 (0.045)	0.191 (0.034)	0.004 (0.031)
VCO2 rest	L/min	CTRL	10	0.164 (0.045)	0.171 (0.040)	0.007 (0.027)
VCO2 rest	L/min	INTV	11	0.166 (0.048)	0.176 (0.050)	0.010 (0.027)
VE rest	L/min	CTRL	10	0.362 (0.097)	0.376 (0.087)	0.014 (0.045)
VE rest	L/min	INTV	11	0.353 (0.088)	0.367 (0.076)	0.014 (0.055)
VEmax rest	L/min	CTRL	10	0.563 (0.177)	0.478 (0.147)	-0.085 (0.150)
VEmax rest	L/min	INTV	11	0.469 (0.158)	0.440 (0.103)	-0.029 (0.144)
RER rest	ratio	CTRL	10	0.826 (0.051)	0.836 (0.083)	0.010 (0.073)
RER rest	ratio	INTV	11	0.893 (0.165)	0.927 (0.211)	0.034 (0.084)
Anaerobic threshold	W	CTRL	10	55.000 (15.811)	58.000 (24.290)	3.000 (13.784)
Anaerobic threshold	W	INTV	11	65.909 (7.006)	64.545 (16.801)	-1.364 (17.043)
Workload	W	CTRL	10	81.130 (30.077)	75.410 (26.655)	-5.720 (15.238)
Workload	W	INTV	11	72.864 (17.807)	74.064 (19.238)	1.200 (11.799)
VO2 exercise	L/min	CTRL	10	1.057 (0.359)	1.058 (0.359)	0.002 (0.085)
VO2 exercise	L/min	INTV	11	0.990 (0.273)	1.049 (0.275)	0.058 (0.101)
VCO2 exercise	L/min	CTRL	10	1.109 (0.401)	1.106 (0.375)	-0.003 (0.102)
VCO2 exercise	L/min	INTV	11	1.056 (0.319)	1.085 (0.320)	0.028 (0.126)
VE exercise	L/min	CTRL	10	2.164 (0.755)	2.164 (0.723)	0.000 (0.179)
VE exercise	L/min	INTV	11	2.047 (0.587)	2.132 (0.588)	0.085 (0.223)
VEmax exercise	L/min	CTRL	10	3.146 (1.299)	2.928 (1.453)	-0.218 (0.287)
VEmax exercise	L/min	INTV	11	2.926 (0.789)	2.484 (0.782)	-0.443 (0.342)
RER exercise	ratio	CTRL	10	1.034 (0.100)	1.042 (0.094)	0.008 (0.055)
RER exercise	ratio	INTV	11	1.025 (0.103)	1.058 (0.098)	0.033 (0.050)
