# Supplementary material for: Unlocking the Healthy Aging Potential of Novel NMN-Based Nutritional Formulations: Cardiopulmonary, Cognitive, and Aging-Related Outcomes in a 42-Day Open-Label Study
Source: Nutrients. 2026 Oct 23;18(21):3490. doi: 10.3390/nu18213490 (PMC13765308; doi:10.3390/nu18213490)
Supplement: Supplementary file 1 [file nutrients-18-03490-s001.zip › Supplementary_Table_S2.pdf]

Supplementary Table S2. CogniTrax domain and index results

Values are mean (sample SD), calculated from the supplied participant records. Change = week 6 minus baseline. CTRL and INTV retain the source group definitions. Before/After source measurements are presented as baseline/week 6 according to the reported 42-day clinical protocol.

Outcome	Unit	Group	N	Baseline	Week 6	Change
Neurocognitive index	standard score	CTRL	10	105.400 (11.635)	103.500 (10.586)	-1.900 (8.672)
Neurocognitive index	standard score	INTV	11	101.000 (9.654)	104.091 (7.609)	3.091 (10.144)
Composite memory	standard score	CTRL	10	103.400 (19.057)	98.100 (27.614)	-5.300 (20.543)
Composite memory	standard score	INTV	11	98.545 (24.897)	104.364 (18.790)	5.818 (13.622)
Verbal memory	standard score	CTRL	10	99.800 (18.867)	97.300 (25.578)	-2.500 (22.897)
Verbal memory	standard score	INTV	11	99.545 (29.378)	108.545 (18.981)	9.000 (24.409)
Visual memory	standard score	CTRL	10	105.400 (13.352)	99.800 (19.147)	-5.600 (11.928)
Visual memory	standard score	INTV	11	98.545 (14.936)	99.818 (14.552)	1.273 (7.086)
Cognitive processing speed	standard score	CTRL	10	112.000 (20.292)	109.900 (20.878)	-2.100 (15.695)
Cognitive processing speed	standard score	INTV	11	97.818 (21.358)	103.727 (11.525)	5.909 (25.074)
Reaction time	standard score	CTRL	10	105.000 (14.537)	103.300 (13.267)	-1.700 (5.438)
Reaction time	standard score	INTV	11	103.364 (5.904)	103.545 (6.846)	0.182 (5.564)
Complex attention	standard score	CTRL	10	104.200 (9.601)	103.600 (8.631)	-0.600 (13.134)
Complex attention	standard score	INTV	11	105.182 (11.617)	107.273 (12.947)	2.091 (17.478)
Cognitive flexibility	standard score	CTRL	10	102.400 (12.367)	102.500 (10.212)	0.100 (9.792)
Cognitive flexibility	standard score	INTV	11	99.091 (12.786)	102.364 (13.500)	3.273 (16.924)
Processing speed	standard score	CTRL	10	117.400 (13.583)	115.300 (15.910)	-2.100 (13.788)
Processing speed	standard score	INTV	11	110.000 (11.593)	112.000 (8.319)	2.000 (6.841)
Executive function	standard score	CTRL	10	103.200 (12.682)	102.800 (11.033)	-0.400 (8.618)
Executive function	standard score	INTV	11	98.909 (12.421)	102.091 (13.642)	3.182 (17.028)
Simple attention	standard score	CTRL	10	100.400 (8.592)	99.600 (10.309)	-0.800 (11.631)
Simple attention	standard score	INTV	11	106.091 (3.562)	100.000 (13.943)	-6.091 (14.474)
Motor speed	standard score	CTRL	10	104.200 (22.607)	101.800 (22.764)	-2.400 (14.470)
Motor speed	standard score	INTV	11	90.636 (30.667)	98.182 (13.992)	7.545 (34.431)
