# Supplementary material for: Unlocking the Healthy Aging Potential of Novel NMN-Based Nutritional Formulations: Cardiopulmonary, Cognitive, and Aging-Related Outcomes in a 42-Day Open-Label Study
Source: Nutrients. 2026 Oct 23;18(21):3490. doi: 10.3390/nu18213490 (PMC13765308; doi:10.3390/nu18213490)
Supplement: Supplementary file 1 [file nutrients-18-03490-s001.zip › Supplementary_Table_S3.pdf]

Supplementary Table S3. Participant-level de-identified immune biomarker data with detection-limit flags

CTACK (pg/mL)

Study code	Group	Baseline	Baseline flag	Week 6	Week 6 flag	Change
8072	CTRL	2008.69	Reported	2219.74	Reported	211.05
151327	CTRL	3434.76	Reported	2814.12	Reported	-620.64
560503	CTRL	3706.24	Reported	2989.35	Reported	-716.89
1237771	CTRL	2487.50	Reported	2349.76	Reported	-137.74
1325768	CTRL	3031.27	Reported	1460.25	Reported	-1571.02
1339173	CTRL	4330.85	Reported	3168.92	Reported	-1161.93
1408973	CTRL	2765.57	Reported	2246.11	Reported	-519.46
1557574	CTRL	1476.24	Reported	1752.91	Reported	276.67
1580105	CTRL	2253.43	Reported	2185.23	Reported	-68.20
1615692	CTRL	2904.95	Reported	2796.99	Reported	-107.96
25682	INTV	2573.91	Reported	2870.72	Reported	296.81
556339	INTV	2879.26	Reported	2598.88	Reported	-280.38
991186	INTV	2850.19	Reported	3186.99	Reported	336.80
1281035	INTV	970.15	Reported	1520.96	Reported	550.81
1286653	INTV	2432.82	Reported	2325.52	Reported	-107.30
1306123	INTV	2591.76	Reported	2561.39	Reported	-30.37
1406936	INTV	2715.62	Reported	2544.26	Reported	-171.36
1484690	INTV	2948.61	Reported	4048.03	Reported	1099.42
1520686	INTV	2415.17	Reported	2688.82	Reported	273.65
1580085	INTV	1855.91	Reported	2045.38	Reported	189.47
1599776	INTV	2413.40	Reported	2488.83	Reported	75.43

Supplementary Table S3. Participant-level de-identified immune biomarker data with detection-limit flags

FGF-21 (pg/mL)

Study code	Group	Baseline	Baseline flag	Week 6	Week 6 flag	Change
8072	CTRL	11.99	Reported	17.04	Reported	5.05
151327	CTRL	NA	Below DL	NA	Below DL	NA
560503	CTRL	17.21	Reported	5.22	Reported	-11.99
1237771	CTRL	37.45	Reported	9.82	Reported	-27.63
1325768	CTRL	45.11	Reported	25.30	Reported	-19.81
1339173	CTRL	13.06	Reported	16.18	Reported	3.12
1408973	CTRL	23.89	Reported	20.21	Reported	-3.68
1557574	CTRL	22.43	Reported	16.39	Reported	-6.04
1580105	CTRL	10.73	Reported	5.33	Reported	-5.40
1615692	CTRL	31.69	Reported	74.95	Reported	43.26
25682	INTV	36.05	Reported	4.19	Reported	-31.86
556339	INTV	8.71	Reported	9.08	Reported	0.37
991186	INTV	58.76	Reported	42.98	Reported	-15.78
1281035	INTV	2.45	Reported	8.90	Reported	6.45
1286653	INTV	56.53	Reported	9.08	Reported	-47.45
1306123	INTV	26.27	Reported	8.52	Reported	-17.75
1406936	INTV	22.80	Reported	27.13	Reported	4.33
1484690	INTV	24.44	Reported	57.74	Reported	33.30
1520686	INTV	13.22	Reported	22.25	Reported	9.03
1580085	INTV	NA	Below DL	NA	Below DL	NA
1599776	INTV	30.12	Reported	48.74	Reported	18.62

Supplementary Table S3. Participant-level de-identified immune biomarker data with detection-limit flags

GDF-11 (pg/mL)

Study code	Group	Baseline	Baseline flag	Week 6	Week 6 flag	Change
8072	CTRL	0.31	Reported	NA	Below DL	NA
151327	CTRL	0.65	Reported	NA	Below DL	NA
560503	CTRL	NA	Below DL	NA	Below DL	NA
1237771	CTRL	NA	Below DL	NA	Below DL	NA
1325768	CTRL	NA	Below DL	NA	Below DL	NA
1339173	CTRL	NA	Below DL	NA	Below DL	NA
1408973	CTRL	NA	Below DL	NA	Below DL	NA
1557574	CTRL	NA	Below DL	NA	Below DL	NA
1580105	CTRL	0.68	Reported	NA	Below DL	NA
1615692	CTRL	NA	Below DL	NA	Below DL	NA
25682	INTV	NA	Below DL	NA	Below DL	NA
556339	INTV	8.64	Reported	7.71	Reported	-0.93
991186	INTV	0.14	Reported	NA	Below DL	NA
1281035	INTV	NA	Below DL	NA	Below DL	NA
1286653	INTV	11.24	Reported	7.21	Reported	-4.03
1306123	INTV	0.48	Reported	0.31	Reported	-0.17
1406936	INTV	0.06	Reported	0.68	Reported	0.62
1484690	INTV	NA	Below DL	NA	Below DL	NA
1520686	INTV	NA	Below DL	NA	Below DL	NA
1580085	INTV	NA	Below DL	NA	Below DL	NA
1599776	INTV	NA	Below DL	NA	Below DL	NA

Supplementary Table S3. Participant-level de-identified immune biomarker data with detection-limit flags

GDF-15 (pg/mL)

Study code	Group	Baseline	Baseline flag	Week 6	Week 6 flag	Change
8072	CTRL	887.96	Reported	734.76	Reported	-153.20
151327	CTRL	422.87	Reported	634.47	Reported	211.60
560503	CTRL	962.14	Reported	735.50	Reported	-226.64
1237771	CTRL	1648.71	Reported	1218.79	Reported	-429.92
1325768	CTRL	2313.41	Reported	1659.40	Reported	-654.01
1339173	CTRL	1607.94	Reported	965.83	Reported	-642.11
1408973	CTRL	776.30	Reported	707.50	Reported	-68.80
1557574	CTRL	637.46	Reported	771.70	Reported	134.24
1580105	CTRL	473.56	Reported	432.33	Reported	-41.23
1615692	CTRL	847.73	Reported	831.86	Reported	-15.87
25682	INTV	1287.54	Reported	1080.62	Reported	-206.92
556339	INTV	679.67	Reported	781.86	Reported	102.19
991186	INTV	1273.08	Reported	1487.01	Reported	213.93
1281035	INTV	381.31	Reported	486.17	Reported	104.86
1286653	INTV	955.19	Reported	852.54	Reported	-102.65
1306123	INTV	1098.77	Reported	1130.22	Reported	31.45
1406936	INTV	713.91	Reported	887.32	Reported	173.41
1484690	INTV	1582.16	Reported	2102.07	Reported	519.91
1520686	INTV	814.09	Reported	827.16	Reported	13.07
1580085	INTV	596.74	Reported	903.88	Reported	307.14
1599776	INTV	1105.62	Reported	1151.00	Reported	45.38

Supplementary Table S3. Participant-level de-identified immune biomarker data with detection-limit flags

GnRH (pg/mL)

Study code	Group	Baseline	Baseline flag	Week 6	Week 6 flag	Change
8072	CTRL	10.88	Reported	NA	Below DL	NA
151327	CTRL	1.81	Reported	NA	Below DL	NA
560503	CTRL	NA	Below DL	NA	Below DL	NA
1237771	CTRL	NA	Below DL	NA	Below DL	NA
1325768	CTRL	NA	Below DL	NA	Below DL	NA
1339173	CTRL	NA	Below DL	NA	Below DL	NA
1408973	CTRL	NA	Below DL	NA	Below DL	NA
1557574	CTRL	NA	Below DL	NA	Below DL	NA
1580105	CTRL	19.58	Reported	24.12	Reported	4.54
1615692	CTRL	NA	Below DL	NA	Below DL	NA
25682	INTV	NA	Below DL	NA	Below DL	NA
556339	INTV	157.69	Reported	148.28	Reported	-9.41
991186	INTV	NA	Below DL	NA	Below DL	NA
1281035	INTV	NA	Below DL	NA	Below DL	NA
1286653	INTV	152.99	Reported	151.42	Reported	-1.57
1306123	INTV	NA	Below DL	NA	Below DL	NA
1406936	INTV	19.58	Reported	19.58	Reported	0.00
1484690	INTV	NA	Below DL	NA	Below DL	NA
1520686	INTV	NA	Below DL	NA	Below DL	NA
1580085	INTV	NA	Below DL	NA	Below DL	NA
1599776	INTV	NA	Below DL	9.83	Reported	NA

Supplementary Table S3. Participant-level de-identified immune biomarker data with detection-limit flags

IL-6 (pg/mL)

Study code	Group	Baseline	Baseline flag	Week 6	Week 6 flag	Change
8072	CTRL	0.86	Reported	0.13	Reported	-0.73
151327	CTRL	1.98	Reported	0.71	Reported	-1.27
560503	CTRL	NA	Below DL	0.41	Reported	NA
1237771	CTRL	2.99	Reported	1.33	Reported	-1.66
1325768	CTRL	1.02	Reported	NA	Below DL	NA
1339173	CTRL	3.33	Reported	4.38	Reported	1.05
1408973	CTRL	NA	Below DL	NA	Below DL	NA
1557574	CTRL	NA	Below DL	0.37	Reported	NA
1580105	CTRL	4.68	Reported	2.94	Reported	-1.74
1615692	CTRL	NA	Below DL	NA	Below DL	NA
25682	INTV	NA	Below DL	NA	Below DL	NA
556339	INTV	NA	Below DL	NA	Below DL	NA
991186	INTV	0.86	Reported	0.01	Reported	-0.85
1281035	INTV	0.27	Reported	NA	Below DL	NA
1286653	INTV	4.73	Reported	2.32	Reported	-2.41
1306123	INTV	1.66	Reported	0.71	Reported	-0.95
1406936	INTV	NA	Below DL	NA	Below DL	NA
1484690	INTV	NA	Below DL	NA	Below DL	NA
1520686	INTV	NA	Below DL	2.44	Reported	NA
1580085	INTV	NA	Below DL	NA	Below DL	NA
1599776	INTV	NA	Below DL	2.18	Reported	NA

Supplementary Table S3. Participant-level de-identified immune biomarker data with detection-limit flags

IL-10 (pg/mL)

Study code	Group	Baseline	Baseline flag	Week 6	Week 6 flag	Change
8072	CTRL	25.05	Reported	25.05	Reported	0.00
151327	CTRL	32.88	Reported	28.93	Reported	-3.95
560503	CTRL	13.98	Reported	21.26	Reported	7.28
1237771	CTRL	41.01	Reported	30.90	Reported	-10.11
1325768	CTRL	23.14	Reported	17.56	Reported	-5.58
1339173	CTRL	13.98	Reported	13.98	Reported	0.00
1408973	CTRL	NA	Below DL	3.25	Reported	NA
1557574	CTRL	NA	Below DL	3.25	Reported	NA
1580105	CTRL	35.29	Reported	22.49	Reported	-12.80
1615692	CTRL	NA	Below DL	NA	Below DL	NA
25682	INTV	21.26	Reported	17.56	Reported	-3.70
556339	INTV	21.26	Reported	30.90	Reported	9.64
991186	INTV	10.53	Reported	17.56	Reported	7.03
1281035	INTV	23.14	Reported	21.26	Reported	-1.88
1286653	INTV	28.93	Reported	28.93	Reported	0.00
1306123	INTV	13.98	Reported	13.98	Reported	0.00
1406936	INTV	NA	Below DL	NA	Below DL	NA
1484690	INTV	NA	Below DL	NA	Below DL	NA
1520686	INTV	NA	Below DL	28.89	Reported	NA
1580085	INTV	NA	Below DL	NA	Below DL	NA
1599776	INTV	NA	Below DL	NA	Below DL	NA

Supplementary Table S3. Participant-level de-identified immune biomarker data with detection-limit flags

IL-18 (ng/mL)

Study code	Group	Baseline	Baseline flag	Week 6	Week 6 flag	Change
8072	CTRL	18.91	Reported	16.45	Reported	-2.46
151327	CTRL	10.99	Reported	14.04	Reported	3.05
560503	CTRL	8.02	Reported	11.33	Reported	3.31
1237771	CTRL	50.16	Reported	32.32	Reported	-17.84
1325768	CTRL	58.75	Reported	62.50	Reported	3.75
1339173	CTRL	8.51	Reported	39.63	Reported	31.12
1408973	CTRL	24.08	Reported	21.72	Reported	-2.36
1557574	CTRL	27.18	Reported	47.90	Reported	20.72
1580105	CTRL	17.02	Reported	10.24	Reported	-6.78
1615692	CTRL	47.52	Reported	61.24	Reported	13.72
25682	INTV	18.55	Reported	14.73	Reported	-3.82
556339	INTV	NA	Below DL	NA	Below DL	NA
991186	INTV	90.52	Reported	109.37	Reported	18.85
1281035	INTV	4.54	Reported	21.92	Reported	17.38
1286653	INTV	NA	Below DL	NA	Below DL	NA
1306123	INTV	5.78	Reported	15.24	Reported	9.46
1406936	INTV	23.72	Reported	17.20	Reported	-6.52
1484690	INTV	30.11	Reported	33.79	Reported	3.68
1520686	INTV	NA	Below DL	NA	Below DL	NA
1580085	INTV	NA	Below DL	NA	Below DL	NA
1599776	INTV	NA	Below DL	9.18	Reported	NA

Supplementary Table S3. Participant-level de-identified immune biomarker data with detection-limit flags

Leptin (pg/mL)

Study code	Group	Baseline	Baseline flag	Week 6	Week 6 flag	Change
8072	CTRL	20760.45	Reported	14813.25	Reported	-5947.20
151327	CTRL	2897.84	Reported	5352.64	Reported	2454.80
560503	CTRL	2085.05	Reported	3156.32	Reported	1071.27
1237771	CTRL	2734.57	Reported	3085.55	Reported	350.98
1325768	CTRL	22097.55	Reported	16767.00	Reported	-5330.55
1339173	CTRL	6961.97	Reported	4393.64	Reported	-2568.33
1408973	CTRL	14809.57	Reported	11639.69	Reported	-3169.88
1557574	CTRL	5560.41	Reported	9086.02	Reported	3525.61
1580105	CTRL	1698.94	Reported	1224.73	Reported	-474.21
1615692	CTRL	14013.95	Reported	23442.70	Reported	9428.75
25682	INTV	2016.29	Reported	4362.79	Reported	2346.50
556339	INTV	19069.51	Reported	17094.28	Reported	-1975.23
991186	INTV	14745.46	Reported	17422.69	Reported	2677.23
1281035	INTV	6280.27	Reported	10965.88	Reported	4685.61
1286653	INTV	5352.64	Reported	3778.05	Reported	-1574.59
1306123	INTV	13709.16	Reported	11445.67	Reported	-2263.49
1406936	INTV	7068.77	Reported	8002.11	Reported	933.34
1484690	INTV	9690.58	Reported	11261.93	Reported	1571.35
1520686	INTV	9993.91	Reported	11064.93	Reported	1071.02
1580085	INTV	1552.23	Reported	1291.94	Reported	-260.29
1599776	INTV	1692.03	Reported	1269.65	Reported	-422.38
