# Supplementary material for: Unlocking the Healthy Aging Potential of Novel NMN-Based Nutritional Formulations: Cardiopulmonary, Cognitive, and Aging-Related Outcomes in a 42-Day Open-Label Study
Source: Nutrients. 2026 Oct 23;18(21):3490. doi: 10.3390/nu18213490 (PMC13765308; doi:10.3390/nu18213490)
Supplement: Supplementary file 1 [file nutrients-18-03490-s001.zip › Supplementary_Table_S4.pdf]

Supplementary Table S4. BLSA functional-age transformation slopes and participant-level derived values

S4a. Transformation slopes

Source metric	Slope as reported	Participant records
VO2	-0.015	21
Workload	-1.5	21
AT	-1	21
VM	-0.6	21
CPS	-0.7	21
EF	-0.5	21
CA	-0.5	21
GDF15	20	21
CTACK	25	21
IL6	0.08	21
IL10	-0.3	21

Supplementary Table S4 (continued)

S4b. VO2

Study code	Group	Baseline raw	Week 6 raw	Raw change source	Baseline age gap source	Week 6 age gap source	Age-gap change source
8072	CTRL	0.388	0.529	0.141	47.467	38.067	-9.4
151327	CTRL	0.768	0.739	-0.029	22.133	24.067	1.933
560503	CTRL	1.275	1.247	-0.028	-11.667	-9.8	1.867
1237771	CTRL	1.176	1.1	-0.076	-5.067	0	5.067
1325768	CTRL	1.611	1.715	0.104	-34.067	-41	-6.933
1339173	CTRL	0.7	0.687	-0.013	26.667	27.533	0.867
1408973	CTRL	1.087	1.087	0	0.867	0.867	0
1557574	CTRL	1.021	1.052	0.031	5.267	3.2	-2.067
1580105	CTRL	1.416	1.458	0.042	-21.067	-23.867	-2.8
1615692	CTRL	1.124	0.971	-0.153	-1.6	8.6	10.2
25682	INTV	1.416	1.574	0.158	-21.067	-31.6	-10.533
556339	INTV	1.037	1.008	-0.029	4.2	6.133	1.933
991186	INTV	1.035	1.077	0.042	4.333	1.533	-2.8
1281035	INTV	0.895	0.872	-0.023	13.667	15.2	1.533
1286653	INTV	0.93	0.936	0.006	11.333	10.933	-0.4
1306123	INTV	0.823	0.997	0.174	18.467	6.867	-11.6
1406936	INTV	0.622	0.596	-0.026	31.867	33.6	1.733
1484690	INTV	1.119	1.075	-0.044	-1.267	1.667	2.933
1520686	INTV	1.02	1.097	0.077	5.333	0.2	-5.133
1580085	INTV	0.568	0.835	0.267	35.467	17.667	-17.8
1599776	INTV	1.426	1.467	0.041	-21.733	-24.467	-2.733

Supplementary Table S4 (continued)

S4b. Workload

Study code	Group	Baseline raw	Week 6 raw	Raw change source	Baseline age gap source	Week 6 age gap source	Age-gap change source
8072	CTRL	40	40	0	26.667	26.667	0
151327	CTRL	40	43.4	3.4	26.667	24.4	-2.267
560503	CTRL	114.4	70	-44.4	-22.933	6.667	29.6
1237771	CTRL	85	78.7	-6.3	-3.333	0.867	4.2
1325768	CTRL	119.2	115	-4.2	-26.133	-23.333	2.8
1339173	CTRL	55	55	0	16.667	16.667	0
1408973	CTRL	70	70	0	6.667	6.667	0
1557574	CTRL	84.9	97	12.1	-3.267	-11.333	-8.067
1580105	CTRL	117.9	115	-2.9	-25.267	-23.333	1.933
1615692	CTRL	84.9	70	-14.9	-3.267	6.667	9.933
25682	INTV	87.4	97.7	10.3	-4.933	-11.8	-6.867
556339	INTV	70	70	0	6.667	6.667	0
991186	INTV	85	70	-15	-3.333	6.667	10
1281035	INTV	70	55	-15	6.667	16.667	10
1286653	INTV	70	55	-15	6.667	16.667	10
1306123	INTV	58	70	12	14.667	6.667	-8
1406936	INTV	70	70	0	6.667	6.667	0
1484690	INTV	70	70	0	6.667	6.667	0
1520686	INTV	70	84.9	14.9	6.667	-3.267	-9.933
1580085	INTV	40	55	15	26.667	16.667	-10
1599776	INTV	111.1	117.1	6	-20.733	-24.733	-4

Supplementary Table S4 (continued)

S4b. AT

Study code	Group	Baseline raw	Week 6 raw	Raw change source	Baseline age gap source	Week 6 age gap source	Age-gap change source
8072	CTRL	40	40	0	30	30	0
151327	CTRL	40	25	-15	30	45	15
560503	CTRL	55	70	15	15	0	-15
1237771	CTRL	55	55	0	15	15	0
1325768	CTRL	40	40	0	30	30	0
1339173	CTRL	40	55	15	30	15	-15
1408973	CTRL	55	55	0	15	15	0
1557574	CTRL	70	70	0	0	0	0
1580105	CTRL	85	115	30	-15	-45	-30
1615692	CTRL	70	55	-15	0	15	15
25682	INTV	70	55	-15	0	15	15
556339	INTV	55	55	0	15	15	0
991186	INTV	70	70	0	0	0	0
1281035	INTV	70	55	-15	0	15	15
1286653	INTV	70	55	-15	0	15	15
1306123	INTV	55	70	15	15	0	-15
1406936	INTV	70	70	0	0	0	0
1484690	INTV	70	40	-30	0	30	30
1520686	INTV	70	85	15	0	-15	-15
1580085	INTV	55	55	0	15	15	0
1599776	INTV	70	100	30	0	-30	-30

Supplementary Table S4 (continued)

S4b. VM

Study code	Group	Baseline raw	Week 6 raw	Raw change source	Baseline age gap source	Week 6 age gap source	Age-gap change source
8072	CTRL	97	90	-7	5	16.667	11.667
151327	CTRL	109	103	-6	-15	-5	10
560503	CTRL	74	115	41	43.333	-25	-68.333
1237771	CTRL	118	118	0	-30	-30	0
1325768	CTRL	115	87	-28	-25	21.667	46.667
1339173	CTRL	81	106	25	31.667	-10	-41.667
1408973	CTRL	112	109	-3	-20	-15	5
1557574	CTRL	68	30	-38	53.333	116.667	63.333
1580105	CTRL	118	106	-12	-30	-10	20
1615692	CTRL	106	109	3	-10	-15	-5
25682	INTV	118	130	12	-30	-50	-20
556339	INTV	45	106	61	91.667	-10	-101.667
991186	INTV	124	118	-6	-40	-30	10
1281035	INTV	66	76	10	56.667	40	-16.667
1286653	INTV	130	130	0	-50	-50	0
1306123	INTV	75	90	15	41.667	16.667	-25
1406936	INTV	130	130	0	-50	-50	0
1484690	INTV	81	124	43	31.667	-40	-71.667
1520686	INTV	115	94	-21	-25	10	35
1580085	INTV	90	93	3	16.667	11.667	-5
1599776	INTV	121	103	-18	-35	-5	30

Supplementary Table S4 (continued)

S4b. CPS

Study code	Group	Baseline raw	Week 6 raw	Raw change source	Baseline age gap source	Week 6 age gap source	Age-gap change source
8072	CTRL	74	79	5	37.143	30	-7.143
151327	CTRL	118	102	-16	-25.714	-2.857	22.857
560503	CTRL	92	110	18	11.429	-14.286	-25.714
1237771	CTRL	134	132	-2	-48.571	-45.714	2.857
1325768	CTRL	130	95	-35	-42.857	7.143	50
1339173	CTRL	113	106	-7	-18.571	-8.571	10
1408973	CTRL	95	98	3	7.143	2.857	-4.286
1557574	CTRL	118	131	13	-25.714	-44.286	-18.571
1580105	CTRL	138	148	10	-54.286	-68.571	-14.286
1615692	CTRL	108	98	-10	-11.429	2.857	14.286
25682	INTV	108	104	-4	-11.429	-5.714	5.714
556339	INTV	105	105	0	-7.143	-7.143	0
991186	INTV	101	103	2	-1.429	-4.286	-2.857
1281035	INTV	85	89	4	21.429	15.714	-5.714
1286653	INTV	105	105	0	-7.143	-7.143	0
1306123	INTV	104	105	1	-5.714	-7.143	-1.429
1406936	INTV	109	111	2	-12.857	-15.714	-2.857
1484690	INTV	38	117	79	88.571	-24.286	-112.857
1520686	INTV	111	103	-8	-15.714	-4.286	11.429
1580085	INTV	96	79	-17	5.714	30	24.286
1599776	INTV	114	120	6	-20	-28.571	-8.571

Slope: -0.7. All values are as reported; NA denotes a blank source cell. Age-gap changes are model-derived year-equivalent units and do not establish changes in chronological or biological age.

Supplementary Table S4 (continued)

S4b. EF

Study code	Group	Baseline raw	Week 6 raw	Raw change source	Baseline age gap source	Week 6 age gap source	Age-gap change source
8072	CTRL	93	79	-14	14	42	28
151327	CTRL	118	105	-13	-36	-10	26
560503	CTRL	77	87	10	46	26	-20
1237771	CTRL	111	110	-1	-22	-20	2
1325768	CTRL	116	114	-2	-32	-28	4
1339173	CTRL	107	106	-1	-14	-12	2
1408973	CTRL	91	105	14	18	-10	-28
1557574	CTRL	110	111	1	-20	-22	-2
1580105	CTRL	105	106	1	-10	-12	-2
1615692	CTRL	104	105	1	-8	-10	-2
25682	INTV	106	111	5	-12	-22	-10
556339	INTV	75	109	34	50	-18	-68
991186	INTV	109	110	1	-18	-20	-2
1281035	INTV	106	102	-4	-12	-4	8
1286653	INTV	89	103	14	22	-6	-28
1306123	INTV	94	95	1	12	10	-2
1406936	INTV	107	69	-38	-14	62	76
1484690	INTV	106	116	10	-12	-32	-20
1520686	INTV	110	112	2	-20	-24	-4
1580085	INTV	80	88	8	40	24	-16
1599776	INTV	106	108	2	-12	-16	-4

Supplementary Table S4 (continued)

S4b. CA

Study code	Group	Baseline raw	Week 6 raw	Raw change source	Baseline age gap source	Week 6 age gap source	Age-gap change source
8072	CTRL	105	90	-15	-10	20	30
151327	CTRL	118	92	-26	-36	16	52
560503	CTRL	86	99	13	28	2	-26
1237771	CTRL	99	108	9	2	-16	-18
1325768	CTRL	105	96	-9	-10	8	18
1339173	CTRL	115	111	-4	-30	-22	8
1408973	CTRL	96	112	16	8	-24	-32
1557574	CTRL	99	105	6	2	-10	-12
1580105	CTRL	112	112	0	-24	-24	0
1615692	CTRL	107	111	4	-14	-22	-8
25682	INTV	111	115	4	-22	-30	-8
556339	INTV	80	113	33	40	-26	-66
991186	INTV	107	117	10	-14	-34	-20
1281035	INTV	111	108	-3	-22	-16	6
1286653	INTV	98	109	11	4	-18	-22
1306123	INTV	105	101	-4	-10	-2	8
1406936	INTV	113	73	-40	-26	54	80
1484690	INTV	117	117	0	-34	-34	0
1520686	INTV	115	111	-4	-30	-22	8
1580085	INTV	89	99	10	22	2	-20
1599776	INTV	111	117	6	-22	-34	-12

Supplementary Table S4 (continued)

S4b. GDF15

Study code	Group	Baseline raw	Week 6 raw	Raw change source	Baseline age gap source	Week 6 age gap source	Age-gap change source
8072	CTRL	887.96	734.76	-153.2	-5.602	-13.262	-7.66
151327	CTRL	422.87	634.47	211.6	-28.856	-18.276	10.58
560503	CTRL	962.14	735.5	-226.64	-1.893	-13.225	-11.332
1237771	CTRL	1648.71	1218.79	-429.92	32.435	10.94	-21.496
1325768	CTRL	2313.41	1659.4	-654.01	65.671	32.97	-32.701
1339173	CTRL	1607.94	965.83	-642.11	30.397	-1.708	-32.105
1408973	CTRL	776.3	707.5	-68.8	-11.185	-14.625	-3.44
1557574	CTRL	637.46	771.7	134.24	-18.127	-11.415	6.712
1580105	CTRL	473.56	432.33	-41.23	-26.322	-28.383	-2.061
1615692	CTRL	847.73	831.86	-15.87	-7.613	-8.407	-0.794
25682	INTV	1287.54	1080.62	-206.92	14.377	4.031	-10.346
556339	INTV	679.67	781.86	102.19	-16.016	-10.907	5.11
991186	INTV	1273.08	1487.01	213.93	13.654	24.351	10.697
1281035	INTV	381.31	486.17	104.86	-30.934	-25.691	5.243
1286653	INTV	955.19	852.54	-102.65	-2.24	-7.373	-5.132
1306123	INTV	1098.77	1130.22	31.45	4.938	6.511	1.573
1406936	INTV	713.91	887.32	173.41	-14.304	-5.634	8.671
1484690	INTV	1582.16	2102.07	519.91	29.108	55.103	25.996
1520686	INTV	814.09	827.16	13.07	-9.295	-8.642	0.653
1580085	INTV	596.74	903.88	307.14	-20.163	-4.806	15.357
1599776	INTV	1105.62	1151	45.38	5.281	7.55	2.269

Supplementary Table S4 (continued)

S4b. CTACK

Study code	Group	Baseline raw	Week 6 raw	Raw change source	Baseline age gap source	Week 6 age gap source	Age-gap change source
8072	CTRL	2008.69	2219.74	211.05	-19.652	-11.21	8.442
151327	CTRL	3434.76	2814.12	-620.64	37.39	12.565	-24.826
560503	CTRL	3706.24	2989.35	-716.89	48.25	19.574	-28.676
1237771	CTRL	2487.5	2349.76	-137.74	-0.5	-6.01	-5.51
1325768	CTRL	3031.27	1460.25	-1571.02	21.251	-41.59	-62.841
1339173	CTRL	4330.85	3168.92	-1161.93	73.234	26.757	-46.477
1408973	CTRL	2765.57	2246.11	-519.46	10.623	-10.156	-20.778
1557574	CTRL	1476.24	1752.91	276.67	-40.95	-29.884	11.067
1580105	CTRL	2253.43	2185.23	-68.2	-9.863	-12.591	-2.728
1615692	CTRL	2904.95	2796.99	-107.96	16.198	11.88	-4.318
25682	INTV	2573.91	2870.72	296.81	2.956	14.829	11.872
556339	INTV	2879.26	2598.88	-280.38	15.17	3.955	-11.215
991186	INTV	2850.19	3186.99	336.8	14.008	27.48	13.472
1281035	INTV	970.15	1520.96	550.81	-61.194	-39.162	22.032
1286653	INTV	2432.82	2325.52	-107.3	-2.687	-6.979	-4.292
1306123	INTV	2591.76	2561.39	-30.37	3.67	2.456	-1.215
1406936	INTV	2715.62	2544.26	-171.36	8.625	1.77	-6.854
1484690	INTV	2948.61	4048.03	1099.42	17.944	61.921	43.977
1520686	INTV	2415.17	2688.82	273.65	-3.393	7.553	10.946
1580085	INTV	1855.91	2045.38	189.47	-25.764	-18.185	7.579
1599776	INTV	2413.4	2488.83	75.43	-3.464	-0.447	3.017

Supplementary Table S4 (continued)

S4b. IL6

Study code	Group	Baseline raw	Week 6 raw	Raw change source	Baseline age gap source	Week 6 age gap source	Age-gap change source
8072	CTRL	0.86	0.13	-0.73	-14.25	-23.375	-9.125
151327	CTRL	1.98	0.71	-1.27	-0.25	-16.125	-15.875
560503	CTRL	NA	0.41	NA	NA	NA	NA
1237771	CTRL	2.99	1.33	-1.66	12.375	-8.375	-20.75
1325768	CTRL	1.02	NA	-0.02	-12.25	NA	NA
1339173	CTRL	3.33	4.38	1.05	16.625	29.75	13.125
1408973	CTRL	NA	NA	NA	NA	NA	NA
1557574	CTRL	NA	0.37	NA	NA	NA	NA
1580105	CTRL	4.68	2.94	-1.74	33.5	11.75	-21.75
1615692	CTRL	NA	NA	NA	NA	NA	NA
25682	INTV	NA	NA	NA	NA	NA	NA
556339	INTV	NA	NA	NA	NA	NA	NA
991186	INTV	0.86	0.01	-0.85	-14.25	-24.875	-10.625
1281035	INTV	0.27	NA	-0.27	-21.625	NA	NA
1286653	INTV	4.73	2.32	-2.41	34.125	4	-30.125
1306123	INTV	1.66	0.71	-0.95	-4.25	-16.125	-11.875
1406936	INTV	NA	NA	NA	NA	NA	NA
1484690	INTV	NA	NA	NA	NA	NA	NA
1520686	INTV	NA	2.44	NA	NA	NA	NA
1580085	INTV	NA	NA	NA	NA	NA	NA
1599776	INTV	NA	2.18	NA	NA	NA	NA

Supplementary Table S4 (continued)

S4b. IL10

Study code	Group	Baseline raw	Week 6 raw	Raw change source	Baseline age gap source	Week 6 age gap source	Age-gap change source
8072	CTRL	25.05	25.05	0	-16.833	-16.833	0
151327	CTRL	32.88	28.93	-3.95	-42.933	-29.767	13.167
560503	CTRL	13.98	21.26	7.28	20.067	-4.2	-24.267
1237771	CTRL	41.01	30.9	-10.11	-70.033	-36.333	33.7
1325768	CTRL	23.14	17.56	-5.58	-10.467	8.133	18.6
1339173	CTRL	13.98	13.98	0	20.067	20.067	0
1408973	CTRL	NA	3.25	NA	NA	NA	NA
1557574	CTRL	NA	3.25	NA	NA	NA	NA
1580105	CTRL	35.29	22.49	-12.8	-50.967	-8.3	42.667
1615692	CTRL	NA	NA	NA	NA	NA	NA
25682	INTV	21.26	17.56	-3.7	-4.2	8.133	12.333
556339	INTV	21.26	30.9	9.64	-4.2	-36.333	-32.133
991186	INTV	10.53	17.56	7.03	31.567	8.133	-23.433
1281035	INTV	23.14	21.26	-1.88	-10.467	-4.2	6.267
1286653	INTV	28.93	28.93	0	-29.767	-29.767	0
1306123	INTV	13.98	13.98	0	20.067	20.067	0
1406936	INTV	NA	NA	NA	NA	NA	NA
1484690	INTV	NA	NA	NA	NA	NA	NA
1520686	INTV	NA	28.89	NA	NA	NA	NA
1580085	INTV	NA	NA	NA	NA	NA	NA
1599776	INTV	NA	NA	NA	NA	NA	NA
